# Association of calcium/calmodulin-dependent protein kinase kinase1 rs7214723 polymorphism with lung cancer risk in a Chinese population

**DOI:** 10.1042/BSR20170762

**Published:** 2017-08-14

**Authors:** Da Chen, Fangming Zhong, Ye Chen

**Affiliations:** 1 The Cardiothoracic Surgery Department, Hang Zhou Red Cross Hospital, 208 Huanchengdong Road, Hangzhou, Zhejiang, China; 2 Department of Pneumology, The Second Affiliated Hospital of Zhejiang Chinese Medical University, 318 Chaowang Road, Hangzhou, Zhejiang, China

**Keywords:** CAMKK1, lung cancer, molecular epidemiology, polymorphism

## Abstract

Calcium/calmodulin-dependent protein kinase (CAMK) kinase1 (CAMKK1) could specifically recognize and activate CAMK I and IV. Furthermore, the activation of CAMK showed positive correlation in proliferation of lung cancer (LC). In addition, a genome-wide association study (GWAS) has identified rs7214723 (E375G) in the *CAMKK1* gene as a susceptibility locus for LC in the U.K. population. Therefore, we conducted a case–control study involving 320 LC patients and 320 controls to validate this conclusion in a Chinese population. Genotyping was performed using a custom-by-design 48-Plex single nucleotide polymorphism (SNP) Scan™ Kit. Our results indicated that the individuals with CC genotype of rs7214723 polymorphism had the higher risk of LC than those who carried TT genotype. Moreover, CAMKK1 rs7214723 polymorphism showed positive correlation with the elevated risk of LC in the allelic model and recessive model, but not in the dominant model. Stratified analysis further confirmed this significant association in male groups and smokers. In conclusion, CAMKK1 rs7214723 polymorphism may be associated with the increased risk of LC. However, larger studies with more diverse ethnic populations are needed to confirm these results.

## Introduction

Lung cancer (LC) is the leading cause of cancer deaths with high incidence rate [1]. Due to complex biological characteristics, LC is extremely difficult to be diagnosed and treated at early stages [2]. In 2017, 222500 new LC cases and 155870 LC deaths are expected to occur in United States [3]. Tobacco usage, air pollution, exposure to carcinogens, genetic factors, and other factors can cause LC [4,5]. In addition, genetic factors play an important role in modifying an individual’s risk for LC.

The calcium/calmodulin-dependent protein kinase (CAMK) kinase (*CAMKK*) gene is located on chromosome 17p13.2 and has 19 exons. CAMKKs phosphorylate and activate specific downstream protein kinases, including CAMK I, CAMK IV, and 5′-AMP-activated protein kinase (AMPK), which mediates a variety of Ca^2+^ signaling cascades [6]. CAMK I was demonstrated to be associated with the proliferation of LC cell lines [7]. Moreover, Williams et al. [8] demonstrated that the activation of CAMK (CAMK II and CAMK IV) inhibits cell cycle progression in small cell lung carcinoma (SCLC) cells. Furthermore, Hulsmann et al. revealed that the activation of mitogen-activated protein kinase (MAPK) contributes to growth inhibition and apoptosis in human LC cells [9]. There is a wide consensus that CAMKK has been shown to undergo autophosphorylation and contains two isoforms (CAMKK1 and CAMKK2) [10]. Therefore, we guessed that CAMMK1, a member of CAMKK, may play a role in the development of LC indirectly.

Rs7214723, a T to C transition, leads a glutamate (E) to glycine (G) substitution at the amino acid position 375. A genome-wide association study (GWAS) has identified rs7214723 (E375G) polymorphism of *CAMKK1* gene as a susceptibility locus for LC in U.K. Caucasians [11]. Subsequently, Truong et al. [12] tended to replicate this finding, but failed to find significant association between the single nuclear polymorphism (SNP) and LC risk amongst Caucasians and Asians. In 2013, Zhang et al. [13] found that CAMKK1 rs7214723 polymorphism contributes to LC risk in a Chinese population and the T allele of rs7214723 could be viewed as a risk allele for LC. Notably, there were two contradictory findings in the Asian populations. Therefore, we conducted a hospital-based study with 320 cases and 320 controls to validate the role of CAMKK1 rs7214723 polymorphism in modifying the risk of LC.

## Methods

### Study subjects

A total of 320 patients diagnosed with LC were consecutively recruited from the Second Affiliated Hospital of Zhejiang Chinese Medical University, between September 2014 and October 2016. The subjects with family history of cancer and biochemical abnormalities did not conform to our inclusion criteria. The healthy controls were free of LC and recruited from the same institutions during the same time period. They were frequency-matched (1:1) to the LC cases based on sex and age (±5 years). A detailed questionnaire related to smoking habits was completed for each patient and control by a trained interviewer. Informed consent was obtained from all the patients and controls prior to their participation. The protocol for the present study was approved by the Ethics Committee of the Second Affiliated Hospital of Zhejiang Chinese Medical University (Hangzhou, Zhejiang, China).

### DNA extraction and genotyping

To investigate the polymorphism of CAMKK1, all study participants provided 2 ml peripheral blood in EDTA tubes and stored at –80 °C until use. DNA was extracted by using the QIAamp DNA Blood Mini Kit (Qiagen, Hilden, Germany). SNP genotyping was performed using a custom-by-design 48-Plex SNP Scan™ Kit (Genesky Biotechnologies Inc., Shanghai, China), which has been described in previous case–control studies [14,15]. This kit was developed according to patented SNP genotyping technology by Genesky Biotechnologies Inc., which was based on double ligation and multiplex fluorescence PCR. For quality control, repeated analyses were done for 4% of randomly selected samples with high DNA quality.

### Statistical analysis

Hardy–Weinberg equilibrium (HWE) for CAMKK1 genotype distributions in controls was tested by a goodness-of-fit chi-squared test. The demographic and clinical characteristics of study participants were evaluated by using the chi-squared test. Odds ratios (ORs) and 95% confidence intervals (CIs) were used to estimate the association between CAMKK1 gene polymorphisms and risk of LC by logistic regression analyses. The most common homozygote was seen as a reference group. All statistical analyses were performed using the SPSS ver 22.0 software package. *P*<0.05 was considered to indicate a significant difference.

## Results

### The characteristics of the study population

The characteristics of LC cases and healthy controls in the present study were summarized in Table 1. There were no significant differences in age, sex, and smoking status between the two groups. According to the tumor histology, 56.3% of LC cases had squamous cell carcinomas.

**Table 1:** Patient demographics and risk factors in LC

Variable	Cases (*n*=320)	Controls (*n*=320)	*P*
Age (years)			
≤50	122	102	0.369
>50	198	218	
Sex			
Female	70 (21.9%)	75 (23.4%)	0.495
Male	250 (78.1%)	245 (76.6%)	
Smoking status			
Non-smoker	65 (20.3%)	78 (24.4%)	0.217
Smoker	255 (79.7%)	242 (75.6%)	
Histology			
Squamous cell carcinoma	180 (56.3%)	–	
Adenocarcinoma	94 (29.4%)	–	
Others	46 (14.4%)	–	

### Association between CAMKK1 rs7214723 polymorphism and LC risk

The frequencies of the genotypes for rs7214723 polymorphism in cases and controls are shown in Table 2. CAMKK1 genotype distribution for rs7214723 polymorphism in the controls conformed to the HWE. In addition, we found that the risk of LC in individuals with CC genotype was 1.8-times higher than that of individuals with TT genotype (CC compared with TT, OR: 1.80; 95% CI: 1.13–2.87; *P*=0.014). The C allele of rs7214723 polymorphism was associated with the increased risk of LC. Furthermore, a significant association was observed in the recessive model (*P*=0.012) and allelic model (*P*=0.023), not in the dominant model (*P*=0.214).

**Table 2:** Logistic regression analysis of association between CAMKK1 rs7214723 polymorphism and risk of LC

Genotype	Cases* (*n*=320)		Controls* (*n*=320)		OR (95% CI)	*P*
	*n*	%	*n*	%		
TC compared with TT	122/133	38.1/41.6	130/149	40.6/46.6	1.05 (0.75–1.48)	0.773
CC compared with TT	61/133	19.1/41.6	38/149	11.9/46.6	**1.80 (1.13–2.87)**	0.014
CC compared with TC compared with TT						
TC + CC compared with TT	183/133	57.2/41.6	168/149	52.5/46.6	1.22 (0.89–1.67)	0.214
CC compared with TC + TT	61/255	19.1/79.9	38/279	11.9/87.2	**1.76 (1.13–2.73)**	0.012
C compared with T	244/388	38.1/60.6	206/428	32.2/66.9	**1.31 (1.04–1.65)**	0.023

Bold values are statistically significant (*P*<0.05).

* The genotyping was successful in 316 cases and 317 controls.

Stratified analyses were conducted according to sex, age, and smoking status (Table 3). For male participants, CAMKK1 rs7214723 polymorphism showed positive correlation with the increased risk of LC in the additive model (CC compared with TT, OR: 1.75; 95% CI: 1.03–2.96; *P*=0.038) and recessive model (CC compared with TC + TT, OR: 1.85; 95% CI: 1.14–2.98; *P*=0.012). The significance also held true for smokers, while there was no significant association in the subgroup analysis of age.

**Table 3:** Stratified analyses between CAMKK1 rs72114723 polymorphism and the risk of LC

Variable	CAMKK1 rs72114723 (case/control)			CC compared with TT	CC + TC compared with TT	CC compared with TC + TT
	CC	TC	TT			
Sex						
Male	54/32	105/120	87/90	**1.75 (1.03–2.96)**; 0.038	1.08 (0.75–1.57); 0.675	**1.85 (1.14–2.98)**; 0.012
Female	7/6	17/10	46/59	1.50 (0.47–4.76); 0.495	1.92 (0.92–4.04); 0.083	1.28 (0.41–4.01); 0.674
Age (years)						
<50	27/14	46/38	48/50	2.48 (0.72–8.44); 0.153	1.46 (0.86–2.49);0.162	1.81 (0.89–3.67); 0.102
≥50	34/24	76/92	85/99	4.39 (0.90, 21.50); 0.067	1.10 (0.75–1.63); 0.617	1.68 (0.96–2.95); 0.071
Smoking status						
Non-smoker	5/8	10/7	66/63	0.60 (0.19–1.92); 0.387	0.96 (0.43–2.11); 0.909	0.58 (0.18–1.84); 0.352
Smoker	56/30	112/123	67/86	**2.40 (1.39–4.14)**; 0.002	1.41 (0.96–2.08); 0.082	**2.18 (1.34–3.55)**; 0.002

Bold values are statistically significant (*P*<0.05).

## Discussion

In the present study, we found CAMKK1 rs7214723 polymorphism was associated with the increased risk of LC in a Chinese population under the allelic model, additive model, and recessive model. Stratified analyses further confirmed this association in the subgroup of male subjects and smokers.

CAMKK1 plays important roles in many aspects, such as regulation of skeletal muscle glucose uptake [16], brain development [17], nucleotide binding [11], and kinase activity [18]. CAMKK1 showed a high degree of substrate specificity, recognizing not only specific amino acid sequences but also the native conformation of CAMK IV and I [18]. It is worth mentioning that the activation of CAMK (CAMK II and CAMK IV) inhibits cell cycle progression in SCLC cells [8]. Our results revealed that CAMKK1 rs7214723 polymorphism increased the risk of LC. Moreover, rs7214723 is located on the exon of CAMKK1 and causes a change in amino acids. Therefore, we guessed that the mutant genotype of rs7214723 might influence substrate specificity of CAMKK1 and inhibit specific downstream protein kinases such as CAMK IV and I. The inhibition is beneficial to cell cycle progression, thereby accelerating the proliferation of LC cells.

Previously, three studies have explored the association between CAMKK1 rs7214723 polymorphism and LC risk [11–13]. Rudd et al. [11] first conducted a large-scale GWAS in U.K. Caucasians to identify susceptibility alleles for LC, analyzing 1529 cases and 2707 controls. They provided evidence that CAMKK1 rs7214723 polymorphism increased the risk of LC [11]. Truong et al. [12] also performed an overall analysis between this SNP and LC risk with a total of 8431 LC cases and 11072 controls. No association was obtained in their study. In addition, subgroup analysis of ethnicity also did not observe an association between rs7214723 polymorphism and LC risk amongst Caucasians and Asians [12]. It is noteworthy that the sample size of Asian groups only consisted of 155 cases and 222 controls, suggesting that we should interpret the data with caution. However, a subsequent Chinese study with 961 LC cases and 999 controls by Zhang et al. [13] indicated that CAMKK1 gene rs7214723 polymorphism increased the risk of LC. They found that TC genotype carriers increased the risk of LC [13]. In the present study, we found CC genotype carriers were associated with an increased risk of LC. Furthermore, we revealed that C allele is a risk allele in lung carcinogenesis. In the study of Zhang et al. [13], they indicated that T allele is a risk allele for LC. Nevertheless, no significant association was found for T or C allele in the data of Table 2. Actually, the T allele was not a risk allele according to their findings. In the stratified analyses, we uncovered positive findings amongst male and smoker groups, which were in accordance with those of Zhang et al. [13]. No significant association was identified in the stratified analysis of age in the present study. To summarize, we think that a meta-analysis for CAMKK1 rs7214723 polymorphism is urgently needed, as the findings of above studies are conflicting. The potential limitations of the present study merit careful consideration. First, this was a hospital-based case–control study; therefore, a selection bias was unavoidable and the subjects are not fully representative of the general population. Second, the polymorphisms investigated, which were based on functional considerations, may not offer a comprehensive view of the genetic variability of CAMKK1. Third, we cannot conduct cell and animal experiments due to limited resources. Finally, the sample size was relatively small, thus the present study may be underpowered.

In summary, mutant genotypes of CAMKK1 rs7214723 polymorphism may be associated with the risk of LC susceptibility, especially amongst males and smokers. LC is a multifactorial disease, and thus contribution of a single SNP is limited. Large-studies are warranted to determine whether CAMKK1 rs7214723 polymorphism is associated with LC risk.

## Abbreviations

CAMK: calcium/calmodulin-dependent protein kinase
CAMKK1: calcium/calmodulin-dependent protein kinase kinase 1
CI: confidence interval
GWAS: genome-wide association study
HWE: Hardy–Weinberg equilibrium
LC: lung cancer
MAPK: mitogen-activated protein kinase
OR: odds ratio
SCLC: small cell lung carcinoma
SNP: single nucleotide polymorphism
